# Reliability, Validity, and Responsiveness of the Visual Perceptual and Fine Motor Function Tests in School‐Aged Children With Mild Intellectual Disabilities

**DOI:** 10.1155/oti/5560931

**Published:** 2026-05-04

**Authors:** Ying-Chun Chou, Yi-Wen Chen, Yee-Pay Wuang

**Affiliations:** ^1^ Department of Occupational Therapy, Kaohsiung Medical University, Kaohsiung, Taiwan, kmu.edu.tw; ^2^ Department of Rehabilitation Medicine, Kaohsiung Medical University Chung-Ho Memorial Hospital, Kaohsiung, Taiwan, kmu.edu.tw

**Keywords:** fine motor, intellectual disabilities, reliability, responsiveness, validity, visual perception

## Abstract

**Introduction:**

Deficits in visual perception and fine motor performance are common in school‐aged children with mild intellectual disabilities (MID). Assessment tools with sound psychometric properties are essential for occupational therapy practice and research.

**Objectives:**

This study examined the reliability (internal consistency and test‐retest reliability), criterion validity and responsiveness of three commonly used assessments: the Test of Visual Perceptual Skills, Fourth Edition (TVPS‐4), the Jebsen–Taylor Hand Function Test (JTHFT), and the Block and Box Test (BBT) in school‐aged children with MID.

**Methods:**

A total of 234 children with MID (134 females, age range: 73–144 months) were assessed at three time points: two preintervention assessments within 14 days (T1 and T2), and one postintervention assessment (T3) following a 4‐month occupational therapy program.

**Results:**

The TVPS‐4 total score demonstrated excellent reliability and good criterion validity. The minimum detectable change (MDC) was 13.89 and the minimal clinically important difference (MCID) was 19.97. The JTHFT showed excellent reliability and criterion validity for both hands. The MDC was 3.66 and 4.67 s for dominant and nondominant hands; the MCID was 8.55 and 6.98 s, respectively. In contrast, the BBT exhibited only fair test‐retest reliability and weak validity and responsiveness.

**Conclusions:**

The TVPS‐4 and JTHFT demonstrated adequate reliability, validity, and responsiveness at both the group and individual levels, supporting their clinical utility for evaluating intervention outcomes and monitoring meaningful changes in visual–perceptual and hand function among school‐aged children with MID.

This study was approved by the Institutional Review Board of Kaohsiung Medical University Hospital (KMUHIRB‐SV(II)‐20190087).

**Trial Registration:**

ClinicalTrials.gov: NCT05451238.

## 1. Introduction

Intellectual disability (ID) is one of the most common developmental disabilities in school‐aged populations, with the majority of affected individuals classified as having mild intellectual disabilities (MID) [1]. Despite diverse etiologies, the distinct impairments characterizing MID are fine motor control deficits (e.g., manual dexterity [MD]) and visual perceptual dysfunctions [2–4]. Fine motor difficulties may arise from multiple factors related to both cognitive and physical development, whereas visual perceptual deficits may reflect alterations in sensory processing and reduced frontal lobe involvement [5]. Together, impairments in visual perceptual and fine motor performance substantially limit activity participation and academic achievement in school‐aged children with MID. For example, students spend much of the school day engaged in sustained reading, writing, and other desk‐based tasks that rely heavily on intact visual–perceptual‐motor skills [6]. Such dysfunctions may have long‐term consequences, persistently limiting performance across multiple domains of community participation and psychosocial functioning [7].

Timely identification and intervention are crucial for enhancing visual motor integration and promoting successful school performance and daily functioning. Accordingly, reliable and sensitive measures are required to detect subtle changes in both fine motor and visual perceptual functions across repeated measurements in children with MID. The Jebsen–Taylor Hand Function Test (JTHFT) [8] and Box and Block Test (BBT) [9] are briefly and easily administered measures suitable for evaluating manual function in children with limited attention span or cognitive abilities and have been widely used to examine functional outcomes of interventions and the manual capabilities in children with disabilities [10, 11]. Similarly, the Test of Visual Perceptual Skills–Fourth Edition (TVPS‐4) [12] has been extensively applied to evaluate the effectiveness of intervention strategies in children with various developmental disabilities [13]. However, despite their widespread use and previous recommendations [14–17], the reliability, validity, and responsiveness of these three measures have not been systematically examined in children with MID.

In pediatric occupational therapy practice, assessment tools are frequently used not only to describe baseline functional abilities but also to monitor progress and evaluate the effectiveness of intervention programs. Therefore, establishing the psychometric properties of these measures is essential to ensure that observed score changes accurately reflect meaningful changes in a child′s functional performance. In particular, outcome measures used in clinical and school‐based settings should demonstrate adequate stability across repeated administrations, meaningful relationships with related constructs, and the ability to detect clinically important changes following intervention.

Test‐retest reliability is important for determining whether repeated measurements produce stable results over time, particularly in children with MID, whose test performance may vary due to attentional fluctuations, cognitive limitations, and differences in task comprehension. Criterion validity is also necessary to establish whether assessment scores meaningfully correspond to established measures of related visual–perceptual and motor abilities, thereby supporting the interpretability and clinical relevance of these instruments. In addition, responsiveness is a critical psychometric property for outcome measures because clinicians frequently rely on these tools to evaluate intervention effects and to determine whether treatment has resulted in meaningful functional improvement at both the individual and group levels.

Despite the widespread use of the JTHFT, BBT, and TVPS‐4 in pediatric rehabilitation, the extent to which these instruments demonstrate adequate reliability, validity, and responsiveness in children with MID remains unclear. Given the need for a comprehensive evaluation of the psychometric properties of occupational therapy assessment tools, the present study investigated the internal consistency, test‐retest reliability, criterion validity, and responsiveness of the JTHFT, BBT, and TVPS‐4 in school‐aged children with MID.

## 2. Method

### 2.1. Participants

This study was part of a larger longitudinal study and was approved by the Institutional Review Board of Kaohsiung Medical University Hospital (KMUHIRB‐SV (II)‐20190087). Children with MID were recruited from 20 elementary schools and 15 hospitals and rehabilitation clinics. The inclusion criteria were as follows: (1) aged 6–12 years; (2) a diagnosis of mild ID determined by board‐certified physicians; (3) no severe emotional or behavioral disturbances; and (4) participation in an occupational therapy program during this study period. Written informed consent was obtained from the parents or legal guardians of all participants prior to participation.

### 2.2. Measures

#### 2.2.1. JTHFT

The JTHFT is a brief and reliable measure of hand function consisting of seven simulated activities of daily living (ADL): check stacking, page‐turning, feeding, picking up small objects, lifting large and lightweight objects, lifting heavy objects and writing. All tasks are timed and three scores are generated: the object score, writing score, and total score. In the present study, the object scores for the dominant and nondominant hands were analyzed. These scores represent the total time (in seconds) required to complete the six tasks excluding the writing task; lower scores indicate better performance. The JTHFT requires only readily available materials and has demonstrated excellent reliability and acceptable validity in children [8, 18, 19].

#### 2.2.2. BBT

Standardized administration and scoring for the BBT was provided by Mathiowetz and colleague [9]. The participant was seated at a table, facing a rectangular box partitioned into two equal‐sized compartments. A total of 150 wooden blocks (2.5 cm^3^ each) are placed in one compartment. The child is instructed to transfer as many blocks as possible, one at a time, from one compartment to the other within 60 s. The scores reflect the number of blocks transferred; higher scores indicate better gross MD. The BBT has demonstrated strong psychometric properties in adults with various pathologies and acceptable reliability in typically developing children [20, 21].

#### 2.2.3. TVPS‐4

The TVPS‐4 comprises seven subtests with 126 black‐and‐white stimuli presented in an easel‐backed flipbook. The seven subtests are as follows: visual discrimination, visual memory, spatial relationships, form constancy, sequential memory, visual figure‐ground, and visual closure. No copying or other complex motor skills are required to respond to the items. Seven scale scores and one total standard score were used for data analysis in the present study. Although reported as stable across ages and texts, evidence supporting validity remains partial, underscoring the need for continued psychometric evaluation [12].

#### 2.2.4. Movement Assessment Battery for Children–Second Edition (MABC‐2)

The MABC‐2 [22] assesses motor competence in individuals aged 3–16 years across three domains: MD, aiming and catching, and balance. The MD component evaluates the fine motor skills required for tasks involving precise and coordinated hand and finger movements. In the present study, the MD standard scores from Age Bands 2 and 3 were used to establish the criterion validity of both the JTHFT and BBT. The MD component yields standardized scores with a mean of 10 and a standard deviation of 3. The MABC‐2 has demonstrated sound reliability and validity, as well as high responsiveness, in typically developing children and in children with developmental coordination disorder (DCD) [22, 23].

#### 2.2.5. Developmental Test of Visual Perception–Third Edition (DTVP‐3)

The DTVP‐3 [24] comprises five subtests assessing closely related visual–perceptual abilities: eye‐hand coordination, copying, figure‐ground, visual closure, and form constancy. Designed for children aged 4–12 years, the DTVP‐3 has demonstrated established reliability and validity. Accumulated psychometric evidence indicates that the DTVP‐3 provides dependable results and serves as a valid measure of visual–perceptual ability in young children [25]. The test provides three composite scores: visual–motor integration (VMI), motor‐reduced visual perception (MRVP), and general visual perception (GVP). In the present study, the GVP composite score was used to examine the criterion validity of the TVPS‐4 in children with MID.

#### 2.2.6. School Function Assessment (SFA)‐Chinese Version

The SFA [26] is a criterion‐referenced measure designed to evaluate a child′s performance in functional activities that support participation in school‐related tasks from kindergarten through sixth grade. The SFA comprises three sections: participation, task supports, and activity performance. The activity performance section assesses school‐related activities such as mobility and locomotion, tool use, and personal care. In the present study, the Physical Tasks Performance Scale (PTPS) within the activity performance section was selected as an external criterion for determining clinically meaningful change.

The PTPS has been used as an anchor to establish the responsiveness of motor function assessments in children with ID and DCD [23, 27]. The PTPS includes 161 functional activities across 12 domains (e.g., recreation and material use), with items rated on a four‐point scale (1–4) according to the level of performance. Psychometric evidence indicates high interrater reliability (ICCs = 0.94–0.98) and good test‐retest reliability (0.87–0.99). Correct classification rates have been reported as 100% for learning disabilities, 97.6% for physical disabilities, and 90.4% for multiple disabilities [26].

### 2.3. Interventions

Occupational therapists employ a range of evidence‐based practice models to guide interventions aimed at improving children′s visual–perceptual‐motor functions (e.g., eye‐hand coordination). Participants in the present study received a conventional pediatric rehabilitation program delivered twice weekly over 4 months (total dosage: 40 h). The program was individualized for each child and incorporated intervention strategies grounded in the motor learning practice model [28]. Core components of this model included repetitive and mass practice of purposeful, goal‐directed functional activities, along with systematic use of feedback and reinforcement to facilitate skill acquisition.

Handwriting is a common and primary therapeutic goal for school‐aged children with disabilities [29]; accordingly, intensive handwriting practice constituted a major component of the intervention. Following written informed consent, five senior occupational therapists administered the treatment sessions. To ensure treatment fidelity, all therapists received standardized training (a 1‐day training workshop conducted by the principal investigator [PI]) prior to the intervention and followed a structured intervention protocol under the supervision of the PI. Regular meetings were conducted to ensure consistency in intervention delivery.

### 2.4. Procedure

Participants were assessed using the BBT, JTHFT, and TVPS‐4 at three time points. Two preintervention assessments (T1 and T2) were conducted 14 days apart, with no intervention administered between sessions. T1 was completed within the week preceding the 4‐month intervention period. Data from T1 and T2 were used to calculate internal consistency, test–retest reliability, and the minimum detectable change (MDC). A postintervention assessment (T3) was conducted within 1 week after completion of the intervention, and data from T1 and T3 were used to evaluate validity and responsiveness.

All BBT, JTHFT, and TVPS‐4 assessments were administered by the same rater in accordance with standardized procedures outlined in the respective examiner′s manuals. Raters were blinded to the study objectives and to participants′ previous scores when conducting subsequent assessments. The PI, who did not participate in the intervention, administered the MABC‐2 and DTVP‐3 at T1 and T3 to establish criterion validity for the three measures. The PI also completed the PTPS at T1 and T3.

### 2.5. Data Analysis

#### 2.5.1. Reliabilities

##### 2.5.1.1. Internal Consistency of the TVPS‐4

Internal consistency evaluates the degree to which different items within the same test all measure the underlying construct. Cronbach′s alpha (*α*) was calculated using T1 data from all participants to evaluate the internal consistency of the TVPS‐4. Alpha values between 0.70 and 0.90 indicate acceptable to strong internal consistency, with values approaching 0.90 considered strong [30].

##### 2.5.1.2. Test‐Retest Reliability

Test‐retest reliability of the JTHFT, BBT, and TVPS‐4 between T1 and T2 was examined using the intraclass correlation coefficient (ICC) based on a two‐way random‐effects model. ICC values range from 0 to 1, with values < 0.50 indicating poor reliability, 0.50–0.75 moderate reliability, 0.75–0.90 good reliability, and > 0.90 excellent reliability.

Measurement precision was further evaluated using the standard error of measurement (SEM), which estimates the amount of error in repeated measurements. SEM was calculated as SDx$\sqrt{1-\text{ICC}}$, where SD represents the standard deviation of the measure and and ICC represents the reliability coefficient. An SEM value less than half of the standard deviation (SD/2) was considered indicative of acceptable reliability, with smaller SEM values reflecting greater measurement precision [27].

##### 2.5.1.3. MDC [23]

The MDC represents the smallest change that exceeds measurement error. A change in score was interpreted as reflecting a true improvement or decline in performance when it exceeded the MDC of a given instrument. The MDC was calculated as MDC = 1.65 × √2 × *S* *E* *M*, where a *z* value of 1.65 corresponds to a 90% confidence interval for clinically meaningful change at the individual level. For each measure (JTHFT, BBT, and TVPS‐4), the proportion of participants whose change scores exceeded the MDC was determined. An MDC percentage (MDC%) of < 30% was considered indicative of acceptable random measurement error [31].

#### 2.5.2. Criterion‐Related Validity [32]

Concurrent validity of the JTHFT, BBT, and TVPS‐4 was examined by calculating Pearson correlation coefficients (r) between each measure and its corresponding criterion at T1 and T3. The criterion measure for the JTHFT and BBT was the MD component of the MABC‐2 (MD), whereas the criterion for the TVPS‐4 was the GVP composite score of the DTVP‐3. Correlations between scores on the JTHFT, BBT, and TVPS‐4 at T1 and the corresponding criterion measures at T3 were used to evaluate predictive validity. Correlation coefficients were interpreted as follows: < 0.25, low; 0.25–0.50, fair; 0.50–0.75, moderate to good; and > 0.75, good to excellent.

#### 2.5.3. Responsiveness

Responsiveness refers to the ability of a measurement instrument to detect changes in scores when a true change in status has occurred. Because average group‐level effects may not adequately reflect clinically meaningful change at the individual level, the present study evaluated responsiveness for the three measures at both the group and individual levels. Group‐level responsiveness was quantified using effect size (ES) and standardized response mean (SRM), whereas individual‐level responsiveness was examined using the minimal clinically important difference (MCID) and the proportion of participants whose change scores exceed the MCID threshold.

##### 2.5.3.1. ES [33]

ES represents the magnitude of change or the strength of the relationship between variables. Values of 0.20–0.50 indicate a small effect, > 0.50–0.80 a moderate effect, and > 0.80 a large effect. In the present study, ES was calculated by dividing the mean change between T1 and T3 by the standard deviation at T1.

##### 2.5.3.2. SRM [27]

The SRM was calculated as the mean change in scores between T1 and T3 (posttest) divided by the standard deviation of the change scores. Consequently, greater variability in the magnitude of change yields a smaller SRM. A positive SRM indicates improvement, whereas a negative SRM indicates a decline in performance.

##### 2.5.3.3. MCID [27]

Both distribution‐ and anchor‐based approaches were used to estimate the MCID for the three measures. For the distribution‐based method, MCID was defined as one‐half of the standard deviation of the change scores between T1 and T3; changes exceeding this threshold were considered clinically meaningful [33]. For the anchor‐based method, groups of children with MID were compared based on therapists′ ratings of functional change using an external anchor—the therapist‐rated PTPS. Mean PTPS change scores were used to classify participants into an improved group (change score ≥ 1) and a no‐change group (change score 0 to < 1). Anchor‐based MCID was calculated as the difference in mean change scores on the BBT, JTHFT, or TVPS‐4 between the improved and no‐change groups. For each instrument, the proportion of participants whose change scores exceeded the MCID was also determined. To examine the validity of the anchor, a one‐way multivariate analysis of variance (MANOVA) was conducted to test for between‐group differences in change scores on the BBT, JTHFT, and TVPS‐4.

## 3. Results

### 3.1. Demographic Characteristics of Participants

A total of 234 children (100 boys and 134 girls) consented to participate. The.

mean age of the participants was 101 months (range = 73–144 months, SD = 27.3), and the mean IQ was 64.91 (SD = 3.73).

### 3.2. Reliability

Table 1 summarizes the reliability indices for the three visual–perceptual and fine motor measures. The TVPS‐4 total score demonstrated excellent internal consistency. Cronbach′s *α* coefficients ranged from 0.89 to 0.94 across subtests and 0.97 for the total score, indicating adequate homogeneity of item content. Test–retest reliability was also excellent, with ICCs ranging from 0.68 to 0.98 for the subtests and reaching 0.98 for the total score. Measurement precision was acceptable, as the SEM for all subtests and the total score met the predefined criterion (SEM < SD/2). The MDC (90% confidence interval) for the total score was 13.89, indicating that an improvement of more than 14 points is required to be interpreted as a true change beyond measurement error for an individual child with MID. Based on this criterion, 29% of participants demonstrated a meaningful improvement in the TVPS‐4 total score. MDC values for the subtests ranged from 1.17 to 4.38.

**Table 1:** Reliability measures of the JTHFT, BBT, and TVPS‐4 (*n* = 234).

	Baseline measurement						
Test	T1	T2	Cronbach′s *α* ^∗^	ICC (95% CI)	SEM	MDC	MDC %
JTHFT							
DH	20.21 ± 7.02	20.00 ± 9.98	—	0.95 (0.94–0.99)	1.57	3.66	58 (25%)
NDH	33.01 ± 7.07	32.12 ± 31.11	—	0.92 (0.94–0.98)	2.00	4.67	63 (27%)
BBT							
DH	44.33 ± 12.26	43.98 ± 27.85	—	0.74 (0.70–0.76)	6.25	14.57	145 (62%)
NDH	39.25 ± 13.88	40.12 ± 20.28	—	0.72 (0.69–0.80)	7.34	17.11	128 (55%)
TVPS‐4							
DIS	12.33 ± 3.55	12.35 ± 4.87	0.89	0.98 (0.96–0.99)	0.50	1.17	46 (20%)
MEM	11.59 ± 2.98	12.26 ± 3.33	0.91	0.96 (0.94–0.98)	0.60	1.40	58 (25%)
SPA	11.98 ± 3.03	12.18 ± 4.15	0.92	0.96 (0.94–0.99)	0.61	1.42	60 (26%)
CON	13.10 ± 4.44	13.25 ± 2.67	0.94	0.98 (0.96–0.99)	0.63	1.47	50 (21%)
SEQ	11.87 ± 4.12	12.07 ± 3.08	0.93	0.95 (0.95–0.98)	0.92	2.14	70 (30%)
FGR	12.12 ± 4.07	12.58 ± 2.78	0.90	0.97 (0.95–0.98)	0.70	1.63	63(27%)
CLO	11.58 ± 3.33	12.06 ± 3.85	0.89	0.68 (0.65–0.74)	1.88	4.38	98 (42%)
TOTAL	110 ± 42.13	113 ± 30.87	0.97	0.98 (0.96–0.99)	5.96	13.89	68 (29%)

*Note:* Asterisk (∗) denotes Cronbach′s *α* was calculated only for the TVPS‐4 because the JTHFT and BBT are performance‐based tests rather than multi‐item scales.

Abbreviations: BBT‐DH, Box and Block Test–dominant hand; BBT‐NDH, Box and Block Test–nondominant hand; CLO, visual closure; CON, form constancy; DIS, visual discrimination; FGR, visual figure‐ground; JTHFT‐DH, Jebsen–Taylor Hand Function Test–dominant hand; JTHFT‐NDH, Jebsen–Taylor Hand Function Test–nondominant hand; MEM, visual memory; SEQ, sequential memory; SPA, spatial relationships; TVPS‐4, Test of Visual Perceptual Skills (4th ed.).

The JTHFT demonstrated excellent test–retest reliability, with ICCs of 0.95 for the dominant hand and 0.92 for the nondominant hand. SEM values indicated good measurement precision for both hands. The MDC (90% confidence interval) for the JTHFT was 3.66 s for the dominant hand and 4.67 s for the nondominant hand. These MDC values indicate that, for 90% of children with MID, improvements exceeding 4 s (dominant hand) and 5 s (nondominant hand) can be interpreted as true changes beyond measurement error. Based on the MDC criterion, 25% of participants demonstrated meaningful improvement in the dominant hand and 27% in the nondominant hand.

The BBT demonstrated moderate test–retest reliability, with ICCs of 0.74 for the dominant hand and 0.72 for the nondominant hand. SEM values did not indicate acceptable measurement precision for either hand, as all values exceeded SD/2. The MDC (90% confidence interval) for the BBT was 14.57 blocks for the dominant hand and 17.11 blocks for the nondominant hand. These MDC values indicate that, for 90% of children with MID, changes of less than 15 blocks for the dominant hand and 18 blocks for the nondominant hand are likely attributable to measurement error rather than true change. Based on the MDC criterion, 62% and 55% of participants demonstrated meaningful improvement on the dominant and nondominant hands, respectively.

### 3.3. Criterion and Anchor Measure

Descriptive statistics of the criterion and anchor measures were examined to facilitate interpretation of the validity and responsiveness analyses. The mean score of the DTVP‐3 was 72.23 (SD = 10.13), which can be interpreted relative to the normative mean of 100 (SD = 15) for typically developing children. The mean score of the MD component of the MABC‐2 was 5.38 (SD = 1.13), compared with the normative mean of 10 (SD = 3) in typically developing children. The mean score of the PTPS of the SFA was 65.75 (SD = 4.32). Because the SFA is a criterion‐referenced measure, PTPS scores reflect the level of functional performance in school‐related physical tasks rather than comparison with normative data. These measures served as criterion and anchor variables in the analyses of validity and responsiveness.

### 3.4. Criterion‐Related Validity

Correlation coefficients between the JTHFT, BBT, and TVPS‐4 and their respective criterion measure at pretest (T1) and posttest (T3) are presented in Table 2. The TVPS‐4 showed moderate concurrent correlations with the DTVP‐3 at both pretest and posttest. Baseline TVPS‐4 scores further demonstrated excellent predictive validity with DTVP‐3 scores at posttest. No significant correlations were observed between the BBT and its criterion measure at either time point. In contrast, correlations between the JTHFT and the MD component were significant and of moderate magnitude for both the dominant and nondominant hands at pretest and posttest. Moreover, JTHFT scores at T1 demonstrated moderate predictive validity with MD at T3.

**Table 2:** Concurrent and predictive validity of the JTHFT, BBT, and TVPS‐4 (*n* = 234).

Test	MABC‐2 MD	DTVP‐3
**Concurrent validity**		
Pretest		
JTHFT‐DH	0.72 ^∗^	—
JTHFT‐NDH	0.61 ^∗^	—
BBT‐DH	0.36	—
BBT‐NDH	0.33	—
TVPS‐4	—	060 ^∗^
Posttest		
JTHFT‐DH	0.78 ^∗^	—
JTHFT‐NDH	0.75 ^∗^	—
BBT‐DH	0.40	—
BBT‐NDH	0.35	—
TVPS‐4	—	0.65 ^∗^
**Predictive validity**		
JTHFT‐DH	0.75 ^∗^	—
JTHFT‐NDH	0.72 ^∗^	—
BBT‐DH	0.39	—
BBT‐NDH	0.40	—
TVPS‐4	—	0.81 ^∗^

Abbreviations: BBT‐DH, Box and Block Test–dominant hand; BBT‐NDH, Box and Block Test–nondominant hand; DTVP‐3, Developmental Test of Visual Perception (3rd ed.); JTHFT‐DH, Jebsen–Taylor Hand Function Test–dominant hand; JTHFT‐NDH, Jebsen–Taylor Hand Function Test–nondominant hand; MABC‐2 MD, Movement Assessment Battery for Children (2nd ed.), manual dexterity component; TVPS‐4, Test of Visual Perceptual Skills (4th ed.).

^∗^ *p* < 0.05.

### 3.5. Responsiveness

Table 3 summarizes the ES, SRM, and MCID for each measure. For the TVPS‐4 total score, the SRM was 0.82 and the ES was 0.52. Across subtests, ES values ranged from 0.18 to 0.67 and SRM values from 0.62 to 1.22, with the figure–ground and visual closure subtests showing the smallest effects (ES < 0.20). Of the 234 participants, 39% were classified as improved, whereas 61% showed no change.

**Table 3:** Responsiveness statistics for the JTHFT, BBT, and TVPS‐4 (*n* = 234).

Test	Difference score (T1‐T3)	Effect size	SRM	MCID (distribution‐based)	MCID (anchor‐based)	MCID %
Hand function test						
JTHFT						
DH	5.75 ± 5.09	0.82^c^	1.13	8.88	8.55	56 (24%)
NDH	3.54 ± 5.90	0.60^b^	0.60	7.67	6.98	58 (25%)
BBT						
DH	4.34 ± 28.60	0.35^a^	0.15	14.30	13.11	138 (59%)
NDH	3.85 ± 31.10	0.28^a^	0.12	15.55	17.45	117 (50%)
Visual perceptual test						
TVPS‐4						
DIS	2.38 ± 3.76	0.67^b^	0.78	1.88	1.91	49 (21%)
MEM	1.52 ± 2.78	0.58^b^	0.82	1.39	1.43	60 (26%)
SPA	1.52 ± 2.24	0.51^b^	0.84	1.82	1.98	60 (26%)
CON	2.97 ± 2.44	0.67^b^	0.63	1.22	1.56	51 (22%)
SEQ	2.10 ± 4.00	0.59^b^	0.93	2.00	2.22	68 (29%)
FGR	2.93 ± 5.02	0.19^a^	0.68	2.51	2.89	63 (27%)
CLO	2.26 ± 4.34	0.18^a^	1.22	4.67	5.35	117 (50%)
TOTAL	29.91 ± 36.22	0.82^c^	0.52	18.11	19.97	70 (30%)

Abbreviations: BBT‐DH, Box and Block Test–dominant hand; BBT‐NDH, Box and Block Test–nondominant hand; CLO, visual closure; CON, form constancy; DIS, visual discrimination; FGR, visual figure‐ground; JTHFT‐DH, Jebsen–Taylor Hand Function Test–dominant hand; JTHFT‐NDH, Jebsen–Taylor Hand Function Test–nondominant hand; MEM, visual memory; SEQ, sequential memory; SPA, spatial relationships; TVPS‐4, Test of Visual Perceptual Skills (4th ed.).

^a^Small effect size.

^b^Medium effect size.

^c^Large effect size.

The anchor‐based MCID for the TVPS‐4 total score, derived from between‐group differences in change scores, was 19.97, with 30% of participants exceeding this threshold (Table 3). Subtest MCID values ranged from 1.56 to 5.35. Analysis of variance (ANOVA) of mean change scores across the seven subtests revealed significant between‐group differences for all subtests (*p* < 0.001 to *p* = 0.004), with the improved group demonstrating greater gains than the no‐change group.

As shown in Table 3, the JTHFT demonstrated strong responsiveness for the dominant hand (ES = 0.82, SRM = 1.13) and moderate responsiveness for the nondominant hand (ES = 0.50, SRM = 0.60). The anchor‐based MCID values, derived from between‐group differences in change scores, were 8.55 s for the dominant hand and 6.98 s for the nondominant hand, with 24% and 25% of participants, respectively, exceeding these thresholds (Table 3). Independent‐samples *t* tests indicated significant between‐group differences for both the dominant hand (*t* = 14.11, *p* < 0.001) and the nondominant hand (*t* = 21.99, *p* < 0.001), with the improved group demonstrating greater gains than the no‐change group. Distribution‐based MCID estimates were comparable with the anchor‐based values.

The BBT demonstrated small responsiveness for both hands, with an ES of 0.35 and an SRM of 0.15 for the dominant hand and an ES of 0.28 and an SRM of 0.12 for the nondominant hand. The anchor‐based MCID values, derived from between‐group differences in change scores, were 14.30 blocks for the dominant hand and 15.55 blocks for the nondominant hand, with 59% and 50% of participants, respectively, exceeding these thresholds (Table 3). Independent‐samples *t* tests indicated a significant between‐group difference for the dominant hand (*t* = 13.97, *p* < 0.001), with the improved group demonstrating greater gains than the no‐change group. In contrast, no significant between‐group difference was observed for the nondominant hand (*t* = 0.17, *p* = 0.862).

## 4. Discussion

### 4.1. Reliability

The present findings demonstrate that the TVPS‐4 is a reliable instrument for assessing visual–perceptual function in children with MID. The Cronbach′s *α* for the TVPS‐4 total score in our sample (*α* = 0.97) exceeded values reported in stroke (0.92), healthy adults (0.90), and children (0.94) [12, 34, 35], as well as another commonly used visual–perceptual measure for school‐aged children (DTVP‐3; *α* = 0.80) [36]. Test–retest reliability of the TVPS‐4 was excellent for the total score and for most subtests in children with MID, with the exception of the visual closure subtest, which showed the lowest reliability (ICC = 0.68). The ICC for the TVPS‐4 total score in the MID sample was comparable with that reported in typically developing children (0.97) and higher than that observed in adults (0.72), whereas ICCs across subtests were also higher than those reported in healthy counterparts (0.46–0.81) [12, 35]. These findings suggest that the TVPS‐4 provides stable measurements of visual–perceptual abilities in children with MID.

The JTHFT demonstrated excellent test–retest reliability over a 14‐day interval in children with MID, with ICCs comparable with those reported in healthy adults and in children with cerebral palsy [33, 37]. Moreover, ICC values for the JTHFT in the present sample exceeded those reported for typically developing children (0.59–0.82) [38]. Although the BBT has been reported to exhibit excellent test–retest reliability in children with cerebral palsy and in typically developing populations [20, 21, 39], reliability for both the dominant and nondominant hands in the current MID sample was only in the moderate range. The relatively lower reliability of the BBT may reflect greater variability in gross MD performance among children with MID.

Reliability coefficients alone provide limited information regarding the precision of test scores at the individual level, and findings across studies are not directly comparable due to differences in samples, analytic methods, and testing intervals. Accordingly, the SEM was examined as a supplementary index of measurement precision. SEM values for the TVPS‐4 total score and most subtests indicated excellent measurement precision, with the exception of the visual closure subtest, for which SEM exceeded the SD/2 criterion. The SEM estimates further supported the stability of the JTHFT across repeated testing in children with MID [37]. In contrast, SEM values for the BBT on both hands were greater than SD/2, indicating lower measurement precision for assessing hand function in this population. These findings suggest that the TVPS‐4 and JTHFT provide more precise measurements for monitoring individual‐level change in children with MID compared with the BBT.

The MDC values of the TVPS‐4 total score and the subtests were smaller than those reported in other populations [34, 35]. The MDC% values larger than 30% indicate substantial random measurement error [40]. The MDC% values of the total scale and subtests were all smaller than 30% except for the visual closure subtest in this study; the results indicated that the TVPS‐4 could be used as a reliable and sensitive measure for children with MID. The MDC value of the JTHFT in MID was smaller than those reported in typically developing children (5.09) and adults (3.32–10.12) [37, 38], and the MDC percentage was less than 30%. The MDC value of the BBT in MID was substantially larger than those reported in CP with a MDC of six blocks [39], and the MDC percentage was larger than 30%. Although both the JTHFT and BBT were always chosen for evaluating hand functions, the reliability measures suggested that the JTHFT might be more reliable and sensitive in assessing hand function in MID. These findings suggest that the JTHFT may be a more appropriate instrument than the BBT for monitoring changes in hand function in children with MID.

### 4.2. Criterion Validity

The TVPS‐4 demonstrated moderate concurrent correlations and excellent predictive validity with the DTVP‐3. Given that visual–perceptual functions are not routinely assessed in standard evaluations for school‐aged children with MID, the TVPS‐4 may serve as a reliable and valid instrument for the comprehensive evaluation of visual‐perceptual abilities in this population [41]. Therefore, the TVPS‐4 may be particularly useful for identifying visual–perceptual deficits and guiding intervention planning in children with MID.

With respect to hand function, our findings indicate that the JTHFT exhibited good criterion‐related validity in children with MID, whereas the BBT did not. Although both tests are time‐based, the JTHFT incorporates tasks that closely resemble ADL, thereby capturing multiple dimensions of hand function within a functional context. This interpretation is consistent with Fabbri′s systematic review [19], which identified the JTHFT as a more appropriate measure of MD in daily activities for children with disabilities. Compared with fine motor subtests of the MABC‐2 and the Bruininks–Oseretsky Test of Motor Proficiency–Second Edition [42], which also provide efficient assessments for children with MID, the JTHFT offers a more time‐efficient and cost‐effective alternative for evaluating fine motor skills in school and clinical settings where resources may be limited. Therefore, the JTHFT may be particularly useful for assessing functional hand performance in children with MID and for monitoring intervention outcomes in school‐based and clinical rehabilitation settings.

### 4.3. Responsiveness

The relatively small ESs indicate that children with MID did not achieve substantial improvements in figure–ground and visual closure in the present study. Both of these domains require higher‐level mental manipulation processes, suggesting that intervention approaches and school curricula that explicitly target mental manipulation strategies may warrant greater emphasis for this population [43]. Nevertheless, these interpretations should be confirmed through replication in comparable samples receiving longer intervention periods or alternative therapeutic protocols. Although visual closure has been hypothesized to contribute to difficulties in recognizing errors during handwriting performance [29], it was the only TVPS‐4 subtest in this study to demonstrate both lower reliability and smaller ESs. Accordingly, clinicians should exercise caution when using the TVPS‐4 as a sole measure to evaluate or explain handwriting performance in children with MID, as the utility of the TVPS‐4 for predicting handwriting outcomes remains inconclusive [13].

The present findings revealed small ESs for the BBT and moderate ESs for the JTHFT, suggesting that children with MID may require more intensive intervention to achieve clinically meaningful improvements in hand function. Alternatively, the magnitude of these effects may reflect limitations of the intervention approach adopted in the present study. Specifically, the intervention primarily employed commonly used task‐oriented strategies targeting functional deficits in the dominant limb, including handwriting and tool use. Given evidence that children with MID exhibit significant interlimb coupling difficulties and greater impairment in the nondominant limb [44, 45], future interventions may benefit from incorporating therapeutic activities that explicitly require bilateral coordination, such as simulated ADL.

Although MDC values are useful for determining whether observed change exceeds measurement error, they do not indicate the magnitude of change required to represent a clinically important improvement in performance. Accordingly, MCID was used to characterize individual‐level change [46]. In this study, both anchor‐based and distribution‐based approaches were applied to estimate MCID. The anchor‐based MCID reflected therapists′ judgments regarding functional performance and activity participation, and MCID estimates for the JTHFT, BBT, and TVPS‐4 were comparable with those obtained using the distribution‐based method. Importantly, MCID values (clinically meaningful change) exceeded corresponding MDC values (measurement error) for both the JTHFT and TVPS‐4, indicating that these instruments possess sufficient precision to detect meaningful clinical change and intervention effects [34].

## 5. Limitations

To our knowledge, this study is the first to comprehensively examine the reliability and responsiveness of commonly used visual‐perceptual and hand function measures in children with MID using both group‐level and individual‐level indices, thereby providing a clinically relevant framework for interpreting treatment‐related change. Nevertheless, several limitations should be considered when applying these findings to practice. First, the generalizability of the results to individuals with ID across different severity levels or age ranges remains uncertain. Future studies should further investigate both sensitivity and specificity to enhance the clinical interpretability of these measures. In addition, prior evidence suggests that the reliability and responsiveness of the JTHFT and BBT may decline over longer assessment intervals; therefore, further research across both short‐ and long‐term follow‐up periods is warranted. Finally, the absence of a control group limits the extent to which observed improvements can be attributed solely to intervention rather than to maturation or practice effects. Future research using controlled study designs and longer follow‐up periods is needed to further establish the clinical utility of these measures.

## 6. Conclusion

The present study provides comprehensive evidence regarding the psychometric properties of commonly used visual–perceptual and hand function measures in children with MID. The findings indicate that the TVPS‐4 and the JTHFT demonstrate strong reliability, acceptable validity, and adequate responsiveness for assessing functional performance in this population. In particular, both instruments were able to detect clinically meaningful changes at the individual and group levels following intervention. In contrast, the BBT showed comparatively lower measurement precision and responsiveness, indicating potential limitations in its ability to detect functional change in this population.

### 6.1. Clinical Implications and Application

Building on these findings, the TVPS‐4 and JTHFT can be considered appropriate outcome measures for evaluating visual–perceptual abilities and functional hand performance in school‐aged children with MID across clinical and school‐based rehabilitation settings. These instruments can assist occupational therapists in monitoring functional progress, evaluating intervention effectiveness, and making informed clinical decisions regarding treatment planning. In contrast, clinicians should interpret BBT results cautiously when using the test to document treatment‐related change, as its measurement characteristics may limit its sensitivity for detecting individual‐level improvements over time. Overall, the present findings support the use of the TVPS‐4 and JTHFT as practical and evidence‐based assessment tools for guiding intervention and tracking functional outcomes in children with MID.

## Author Contributions

**Ying-Chun Chou:** conceptualization, methodology, writing – original draft, writing – review & editing. **Yee-Pay Wuang:** conceptualization, methodology, writing – review & editing. **Yi-Wen Chen:** investigation, data curation, project administration. All authors participated in the critical review.

## Funding

No funding was received for this manuscript.

## Disclosure

All authors approved the manuscript submission.

## Ethics Statement

This study was carried out in accordance with the Declaration of Helsinki and was approved by the Institutional Review Board of Kaohsiung Medical University Hospital (KMUHIRB‐SV(II)‐20190087).

## Conflicts of Interest

The authors declare no conflicts of interest.

## Data Availability

The data that support the findings of this study are available from the corresponding author upon reasonable request.
